# The “puberty deadline”: cultural constructions of maturity, anticipated shame, and child marriage as a perceived risk-mitigation strategy in rural Niger

**DOI:** 10.3389/frph.2026.1922270

**Published:** 2026-09-03

**Authors:** Jean Christophe Fotso, Aichatou Hamadou, Ferdinard Ngong, Paul Bukuluki

**Affiliations:** 1 EVIHDAF, Yaoundé, Cameroon; 2 EVIHDAF, Niamey, Niger; 3 Makerere University's School of Social Sciences, Kampala, Uganda

**Keywords:** child marriage, gender norms, Niger, puberty, qualitative research

## Abstract

**Introduction:**

In rural Niger, child marriage remains highly prevalent, often functioning as a mechanism of risk mitigation. This qualitative study explores the cultural and bio-social drivers of child marriage, focusing on the intersection of physical maturation, anticipatory shame, and economic vulnerability.

**Methods:**

The study drew on 53 in-depth interviews, six body mapping focus group discussions (FGDs), and 9 social norms mapping FGDs with adolescents, parents, and community leaders (n = 165) in the Maradi region.

**Results:**

We find that participants perceive the biological onset of menarche as a ‘puberty deadline.’ This biological trigger socially reclassifies girls from children to women perceived to be vulnerable, sparking acute parental anxiety over premarital sexuality and the stigma of out-of-wedlock pregnancy. Conversely, the reported covert use of contraceptives by some adolescent girls, sometimes with maternal complicity, serves to protect reputations while delaying marriage, highlighting complex implications for adolescent autonomy and consent.

**Discussion:**

Our findings highlight a stark gender asymmetry in the transition to adulthood and demonstrate that child marriage in this context is perceived to be driven by the dual pressures of moral safeguarding and poverty. Implications for future interventions include addressing both the gendered cultural narratives of maturity and the underlying economic desperation sustaining the practice, which may be essential for achieving lasting change.

## Introduction

Child marriage, defined as a formal marriage or informal union before the age of 18, remains a profound global challenge driven by a complex, context-dependent interplay of gender inequality, the cultural regulation of girls’ sexuality, economic vulnerability, and familial survival strategies (1–5). Globally, an estimated 640 million girls and women alive today were married in childhood, and at the current pace of progress, it will take another 300 years to eliminate the practice (6). Sub-Saharan Africa is emerging as a region of particular concern where one in three girls marry before the age of 18, with progress lagging significantly due to rapid population growth (6–8). The burden falls particularly heavily on West and Central Africa, a region that has made little progress over the past 25 years, with stalled or increasing trends observed in several countries (6, 9). Across sub-Saharan Africa, one in three women aged 20–24 years were first married or in a union before age 18; if current trends continue, the region is projected to account for 41% of the world's child brides by 2030 (3). Within this region, Niger consistently records the highest prevalence rates in the world, with approximately 76%–77% of women aged 20–24 married before their 18th birthday (9, 10). Furthermore, Niger possesses the highest fertility rate globally alongside a high adolescent birth rate, with 27% of adolescent girls experiencing a first birth before turning 18 (9). While the public health, psychosocial, and demographic outcomes associated with child marriage—including early childbearing, maternal mortality, obstetric fistula, and diminished relationship quality—are increasingly recognized (11–13), addressing the practice requires a deeper examination of the lived experiences and how child marriage is understood, negotiated, and reproduced through interacting social, gendered, cultural, and economic processes.

Recent scholarship emphasizes the necessity of applying a cultural lens to understand how adolescence, sexuality, and readiness for marriage are construed within practicing communities (1, 5, 14). Drawing on social norms theory, we conceptualize social norms as informal rules that shape what is considered acceptable behavior within a given context. These norms are sustained within a specific reference group through shared empirical expectations (beliefs about what others do) and normative expectations (beliefs about what others approve of), alongside anticipated social sanctions for non-compliance (5, 15). Within this framework, *gender norms* operate as a critical subset of social norms that influence specific expectations and behaviors based on an individual's gender (5). Importantly, these norms are not strictly deterministic; rather, individuals may comply with, negotiate, or actively resist these normative expectations depending on their intersecting social and economic realities. In many patriarchal, low-resource settings, marriage is not merely an economic transaction but a critical institution for managing adolescent sexuality and reinforcing constructions of adulthood (1, 14, 16). While puberty encompasses a broad developmental process, the specific biological event of menarche frequently acts as the primary definitive marker for socially constructed maturity in this context. This biological trigger initiates a precarious transition period wherein a girl's body becomes the subject of intense community scrutiny and regulation (1, 16). The social meaning attached to female physical maturation dictates a girl's perceived “marriageability,”—defined here as the community's assessment of her eligibility and readiness for marriage (17)—effectively operating as a “puberty deadline” for her entry into adulthood. Because girls are frequently identified with their future roles as wives and mothers rather than providers, life choices in these settings are often reduced to a rigid “either/or” option: continue formal schooling or get married (18, 19). Once a girl is married, it is exceptionally rare for her to remain in school, a discontinuation that severely restricts her future agency and economic opportunities (7, 19).

Consequently, in environments characterized by poverty and limited educational opportunities, parents often rely on child marriage as a culturally approved risk-reduction measure to prevent premarital sex, out-of-wedlock pregnancy, and the ensuing social stigma (5, 14, 20). Driven largely by extreme poverty, illiteracy, and a misconception of the need to “protect” a girl's sexuality, families may also seek “refuge” for their daughters in early marriage during economic crises, conflicts, or climate-related shocks (6, 20). In West Africa specifically, the financial incentives of bride wealth and the desire to alleviate household poverty by transferring the care of female children provide powerful economic motivations for the practice (3, 4, 9). Building on these dynamics, we introduce the analytical construct of the “puberty deadline” to propose our interpretation that parents utilize early marriage as an immediate economic and moral coping mechanism—a practice that ultimately traps girls in an intergenerational cycle of poverty by drastically reducing their lifetime educational attainment and future labor force earnings (8, 19, 21). Understanding child marriage in Niger therefore requires exploring the complex, intersectional dynamics between the biological triggers of puberty, the cultural policing of female sexuality, and the economic realities of rural pastoralist and agricultural communities.

Despite a growing evidence base on the prevalence and drivers of child marriage, three critical gaps remain regarding the sociocultural mechanisms that govern adolescent sexuality and child marriage in Niger.

**First,** while the association between perceived maturity and marriage timing is increasingly recognized (1, 17), there is limited qualitative research in Niger exploring how menarche is interpreted with respect to maturity, marriageability, and gendered transitions to adulthood. There is a need to deconstruct how puberty triggers an acute cultural panic surrounding female sexuality, fundamentally reclassifying girls as “at-risk” while adolescent boys are afforded greater sexual autonomy and a prolonged transition to adulthood (16).

**Second,** current frameworks often describe child marriage as a general consequence of patriarchal norms (15) but fail to adequately explore the specific mechanisms of anticipatory “shame,” specifically within the Niger cultural context. While literature from other African contexts suggests that the threat of premarital pregnancy drives marriage decisions (5, 14), less is known about how this operates as a proactive strategy of risk mitigation orchestrated by parents in rural Niger. Furthermore, the role of maternal agency—such as mothers covertly seeking family planning to protect their daughters’ reputations before marriage—remains heavily underexplored (22).

**Third,** there is a gap in understanding how this cultural fear of shame intersects with economic vulnerability. While poverty is a known driver of child marriage, qualitative research must untangle how the “puberty deadline” is utilized by impoverished families simultaneously as a moral protection mechanism and an economic offloading strategy, thereby heavily constraining young people's agency and life choices (5, 16).

To address these gaps, this paper draws on qualitative data from rural Niger to examine child marriage as a bio-social mechanism for the management of shame. Within this framework, we define *anticipatory shame* as the cultural fear and social stigma associated with premarital female sexuality and out-of-wedlock pregnancy (5, 14, 23). To manage this overarching threat of shame, families employ proactive strategies of *risk mitigation* (14)***—***specifically, the restriction of girls’ mobility, the covert use of contraceptives (22), and ultimately, child marriage (5, 14). By understanding how risk mitigation strategies are deployed to manage shame, this paper aims to achieve the following objectives:

1. **To explore the cultural construction of the “puberty deadline,”** examining how menarche is uniquely gendered and operates as a biological trigger that socially reclassifies adolescent girls from children to marriageable, “at-risk” women.
2. **To analyze the dynamics of anticipatory “shame” and risk mitigation,** investigating how parental anxieties surrounding adolescent female sexuality drive the use of child marriage and covert maternal family planning as distinct risk reduction strategies that shape girls’ physical and social autonomy.
3. **To examine how social norms interplay with structural factors such as poverty to drive risk and vulnerability to early marriage,** highlighting how the normative pressures to protect family honor are compounded by poverty, utilizing preventive child marriage as a dual strategy for moral safeguarding and economic relief.

By achieving these objectives, this study contributes to a more nuanced understanding of how adolescent sexuality and gender inequality are culturally negotiated in the Sahel, providing vital insights for culturally informed reproductive health and gender-transformative programming.

## Materials and methods

### Study design and setting

This study draws on primary qualitative data from a cross-sectional baseline assessment conducted in April and May 2024 as part of the FOUNDATIONS project, a multi-year, multi-country gender-transformative program implemented by Save the Children Canada and funded by Global Affairs Canada, which aims to improve adolescent sexual and reproductive health (SRH) in West Africa. Rather than a secondary analysis, the qualitative data collection instruments—such as body mapping and social norms discussions—were purposefully designed *a priori* to answer the specific research questions of this study. To capture the cultural dimensions of sex, gender, and reproductive readiness, the study utilized a constructivist qualitative design, a method well-suited for examining how communities perceive and construct phenomena such as child marriage and maturity (1, 24).

The study was conducted in the Tessaoua health district within the Maradi region of Niger. Maradi was purposefully selected due to its distinctively high prevalence of child marriage and early childbearing, deeply entrenched patriarchal structures, and high rates of poverty (6, 19). Data were collected across five rural and peri-urban communities (Oura, Maiguije, Magaria Sud, Awache, and Garare). These sites were originally selected for the parent “Foundations” project to capture a gradient of geographic remoteness and access to services. Four communities were designated as future intervention sites, while the fifth (Garare) served as a control site, matched specifically for its social, economic, and health-level similarities. Because this study utilizes the baseline assessment data, all interviews and focus groups were conducted prior to any intervention exposure. Furthermore, a community's status as an intervention or control site did not alter our standardized recruitment or data collection protocols. Finally, while these communities vary in their distance to the district center (ranging from 25 km to 62 km) and some are geographically classified as peri-urban, they share highly similar socio-demographic profiles: they are overwhelmingly Hausa and rely almost exclusively on subsistence agriculture and small-scale livestock trading. Because this agrarian reality deeply entrenches household economic vulnerability and customary law across all five sites, we characterize the overarching study context as functionally rural. This provided a diverse representation of the Hausa-speaking context where customary law, Islamic traditions, and local social norms heavily govern family life and adolescent sexuality (15, 19).

### Study population and sampling

We utilized purposive sampling to recruit a diverse range of participants capable of providing rich insights into the cultural expectations of puberty, community norms surrounding family honor, and adolescent lived experiences. Eligibility criteria varied by participant group: adolescents were required to be aged 10–19 and reside in the selected communities; parents were required to have at least one adolescent child; and community stakeholders were required to hold a recognized leadership or influential role (e.g., village chiefs, religious leaders). Recruitment was facilitated by community gatekeepers—primarily village chiefs—who provided initial introductions and helped identify eligible households. Following these introductions, our trained national research team directly approached potential participants at their homes to privately explain the study and seek informed consent. The study stratified adolescents by age (10–14 and 15–19 years), sex, and marital status to capture distinct developmental stages and vulnerabilities to the “puberty deadline”. The overarching sample composition and target size (*n* = 165) were predetermined by the parent project's baseline assessment protocol to ensure balanced representation across these strata. However, during the subsequent analysis for this study, we evaluated information adequacy and confirmed that this predetermined sample size was robust enough to reach thematic data saturation regarding the core concepts of the “puberty deadline” and anticipatory shame.

Participant recruitment was facilitated through introductory visits with local authorities, including prefects, mayors, and village chiefs. With the support of these local leaders, the research team identified community mobilizers who assisted in recruiting eligible individuals using convenience and snowball sampling techniques.

**Inclusion criteria** required that participants lived in one of the identified study localities, matched the required target profiles (e.g., specific age groups, marital statuses, or community roles), and were willing and able to provide informed consent. For minor participants, both parental consent and the adolescent's individual assent were mandatory for inclusion.

**Exclusion criteria** applied to individuals who were unable to provide informed consent due to severe unavailability or disability, or in cases where parents or guardians refused to allow their minor child to participate.

### Data collection

Data collection was carried out by a team of eight national researchers fluent in local languages (Hausa and French) and trained in sensitive interviewing techniques. To mitigate the potential influence of age, gender, and authority on participant disclosure, all data collection activities were strictly segregated; adolescents, parents, and community leaders participated in completely separate sessions. Furthermore, adolescent focus group discussions (FGDs) were stratified by age (10–14 and 15–19), sex, and marital status to ensure safe and open dialogue.

All interviews and FGDs were conducted by gender-matched pairs of researchers, with one serving as the lead facilitator and the other as the dedicated note-taker. Sessions lasted between 90 and 120 min and took place in private, participant-selected locations to ensure auditory privacy. The topic guides were co-developed by the core research team and local experts and underwent rigorous piloting in a peri-urban neighborhood in Maradi to ensure cultural relevance and sensitivity before deployment. During all sessions, detailed field notes were taken to capture non-verbal cues and group dynamics; these handwritten notes were expanded and digitized immediately following the sessions to complement the audio recordings. To ensure findings were not produced by conceptually predetermined prompts, the data collection guides utilized open-ended, non-directive questioning. Data collection for this study was structured around three qualitative methods, triangulating adolescent and parental perspectives, as described in Table 1:

- **Body mapping focus group discussions (FGDs) with adolescents (*n*** **=** **6 FGDs):** We conducted six FGDs with younger adolescents aged 10–14 (comprising 24 girls and 24 boys in total, ∼8 per group). This interactive, visual tool was critical for eliciting cultural constructions of maturity. Adolescents drew gendered bodies to facilitate discussions on how the onset of physical maturation (such as menarche) is interpreted by the community as a social milestone for sexual readiness, thereby triggering immediate social reclassification and marital eligibility (17). Rather than asking directly about a “deadline”, facilitators utilized projective techniques, asking adolescents to draw a typical peer and prompting: “*What kinds of activities can a girl/boy your age do? What are the differences between boys and girls, and why?”*
- **Social norms mapping FGDs with parents and community leaders (*n*** **=** **9 FGDs):** We conducted nine FGDs with adult community members (24 fathers, 24 mothers, and 16 community stakeholders). Utilizing a community timeline, this guide explored the collective narratives and normative expectations surrounding adolescent female. Parents were asked to reflect broadly on community expectations using open-ended prompts such as: “*What are the current norms regarding child marriage, and what factors influence their perpetuation?”*. It was from these participant-led discussions that the themes of parental fear of shame regarding out-of-wedlock pregnancy, and families justifying preventive child marriage as a moral safeguard, inductively emerged (5, 14).
- **In-Depth Interviews (IDIs) with adolescents (*n*** **=** **53):** We conducted individual interviews with older adolescents (16 married girls, 17 unmarried girls, and 20 unmarried boys). Adopting a “life history” approach (12), these interviews explored personal narratives of the transition to adulthood. Instead of specifically probing for theoretical concepts, the interviews asked adolescents to reflect broadly on cultural pressures to marry early, their experiences of constrained agency, and how poverty and fear of sexual transgression accelerated their marital transitions. It was during the subsequent thematic analysis of these broad narratives that the specific theoretical construct of the “puberty deadline” inductively emerged.

**Table 1:** Distribution of data collection tools by study site.

Data collection tool	Intervention sites				Control (Garare)	Total
	Oura	Maiguije	Magaria	Awache		
IDIs with adolescents (16 married girls, 17 unmarried girls, and 20 unmarried boys)	12	8	8	7	18	53
Body mapping FGDs with adolescents aged 10–14 (24 girls and 24 boys)	1	1	1	1	2	6
Social norms mapping FGDs with adult community members (24 fathers, 24 mothers, and 16 community leaders)	1	2	1	2	3	9

The numbers reported for FGDs represent the number of FGDs. Each FGD consisted of roughly 8 participants, yielding a total of 112 FGD participants. Combined with the 53 IDIs, we have 165 participants in total.

### Data analysis

All interviews and focus group discussions were audio-recorded with participant consent, transcribed verbatim from local languages into French, and imported into MAXQDA for data management**.** Given the constructivist paradigm of the study, data were analyzed using a hybrid thematic analysis approach (25). The analytical process involved multiple auditable stages. First, a preliminary deductive codebook was developed by the core research team based on the Gender and Adolescence: Global Evidence (GAGE) conceptual framework. This codebook was piloted on an initial subset of transcripts, during which inductive codes (such as “anticipatory shame” and the “puberty deadline”) were generated and added to capture emerging, context-specific phenomena.

Coding was conducted in French by a team of trained bilingual qualitative analysts. To ensure analytical rigor, 20% of the transcripts—purposefully selected to represent a cross-section of participant types, genders, and study sites—were independently double-coded. Any coding discrepancies were resolved through regular consensus-building meetings between the coders and the senior research team. Furthermore, transcripts were coded using specific “difference codes” (e.g., age, gender, marital status) to facilitate an intersectional analysis. This intersectional lens was applied to examine how deeply rooted patriarchal narratives, cultural constructions of maturity, and economic vulnerability interacted to govern the sexual and reproductive lives of adolescent girls (22, 26).

To develop the final themes and directly address the study's objectives, the three data sources were systematically triangulated to contrast intergenerational perspectives. Specifically, the normative community pressures identified in the Social Norms FGDs—which illuminated parents’ overarching anxieties regarding family honor and preventive risk-management strategies—and the biological perceptions from the Body Mapping FGDs—which captured adolescents’ perceptions of physical maturation and the biological realities of puberty—were utilized to establish the macro-level cultural context. The micro-level lived experiences from the adolescent IDIs were then nested into this context and compared to understand how these competing bio-social pressures and community expectations constrained individual adolescents’ agency and shaped their marital trajectories (26). Finally, the selected quotations included in this manuscript were translated from French to English by bilingual authors, and independently verified by a second researcher via back-translation checks to ensure cultural and conceptual equivalence.

### Ethical considerations

Ethical approval was granted by the *Comité National d'Ethique pour la Recherche en Santé* (CNERS) in Niger (Reference No. 14/2023/CNERS). Strict informed consent procedures were followed: written informed consent was obtained from all adult participants. For all adolescents under 18, written informed consent was obtained from their legal guardian or next of kin. For unmarried adolescents, this was their biological parent; for married adolescents under 18, consent was provided by their husband (acting as the customary legal guardian) or their parent, depending on their residential arrangement. Crucially, to protect voluntary participation from parental or spousal influence, our trained national researchers sought the adolescent's individual assent privately, entirely separate from the guardian's consent process. All interviews were conducted in private, participant-selected locations out of earshot of family members or husbands, and adolescents were explicitly informed that they could decline participation or terminate the interview at any time without consequence. To protect participants, all data were anonymized, and interviews were conducted in private locations chosen by participants. To further safeguard against deductive identification within these small rural networks, specific community markers and personal details were altered or removed from the transcripts. Furthermore, while facilitators explicitly urged all participants to respect group privacy and keep discussions private, we acknowledge the inherent limitation that absolute confidentiality cannot be guaranteed within focus group settings. Given the highly sensitive nature of discussing cultural taboos such as menarche, premarital sexuality, and the stigma of child marriage, stringent ethical protocols were enforced (John et al., 2019). Furthermore, as part of the broader study protocol, a referral mechanism was established; researchers carried “referral forms” to link any participants disclosing abuse or gender-based violence to local health and child protection services. The study adhered to WHO guidelines for research on violence against women and children, prioritizing the safety and confidentiality of respondents above data collection. Finally, to compensate for their time and any travel inconveniences, participants received a small in-kind token of appreciation (soap valued at approximately 1,000 CFA francs).

## Results

The analysis of qualitative data from rural Niger reveals that participants commonly described child marriage as serving a perceived function for managing social risk and preserving family honor. This management is heavily structured by cultural constructions of physical maturity, intense parental anxiety over premarital sexuality, and intersecting economic vulnerabilities.

### The “puberty deadline”: menarche, secrecy, and social reclassification

Our data indicate that menarche—a visible biological milestone of puberty rather its onset—serves as a critical “deadline” that fundamentally shifts a girl's social status from “child” to “at-risk woman”. In all study sites, participants consistently identified menarche (which they reported typically occurs between ages 13 and 15 in their communities) as the signal that a girl is ready for marriage. Adolescents consistently report early marriage shortly after puberty: “Most often, the girl is given in marriage at 14 or 16” (Body Mapping, Boy, Maiguije). Peers act as a powerful reference group enforcing this timeline; participants reported that girls who delay marriage often face social sanctions, including stigmatization. As adolescent girls explained, failing to marry shortly after puberty results in being labelled an outcast: “Because people will call her an old maid… when all her generation is married. It only remains her alone who is not married” (Body Mapping, Girls, Garare).

However, these early physical transitions are often shrouded in intense secrecy. Mothers and maternal aunts operate as the primary reference groups responsible for managing this transition. They enforce strict hygiene norms so that girls can achieve the positive reward of a preserved social reputation and avoid public shame: “We advise them to be clean and take care of themselves to the point where someone couldn't even understand that they are menstruating” (Social norms, Mothers, Awache). Mothers frequently find it uncomfortable to discuss reproductive health directly with their daughters, often delegating the management of menstruation to maternal aunts according to local social norms. Because of this profound sense of shame, adolescent girls frequently avoid discussing puberty with their own mothers. Illustrating the confusion and embarrassment surrounding these early physical changes, one girl admitted, “I didn't speak to anyone… I am ashamed to talk about it” (IDI, Disable girl, Maiguije). Furthermore, peers act as a secondary reference group that rigidly enforces this secrecy through the threat of negative sanctions. FGD data reveals that younger girls are terrified of their peers discovering their menstruation, fearing immediate social punishment such as public humiliation, gossip, and ostracization. As participants explained, “If the blood comes out, they will mock them and chase them away… as soon as others see that you are bleeding, they will divulge it to others” (Body Mapping, Girls, Garare). Instead of facing potential scrutiny at home or negative sanctions from peers, girls actively seek out judgment-free environments, opting to confide in peers or specific aunts or older “wise women” who offer understanding and the reward of discretion: “By chance, if you meet a wise woman, she will keep the secret and ask you to go change your wrapper” (Body Mapping, Girls, Garare). Highlighting this preference for seeking out non-judgmental family members, another participant explained her choice to confide in her aunt rather than her mother: “…she understood me and made me understand not to worry about it. It is a common thing among all women of puberty age” (IDI, Married girl, Garare). Maternal aunts also operate as a critical reference group guiding girls to adhere to the social norm of arranged marriage immediately following puberty. They encourage compliance by emphasizing the ultimate positive reward: parental blessings. Conversely, challenging this norm by refusing a parent's choice triggers negative sanctions from the broader community and the prospective husband's family. Illustrating this dynamic, one married adolescent recounted:

I had wanted to refuse a forced marriage. I consulted my aunt and she advised me to obey my parents to have their blessings, and I finally accepted… [If a girl refuses] she is insulted in the village, some people say that she disobeys the word of her own parents, and even if she marries the man she claims to love, this girl will have no importance in the eyes of her husband's family members (IDI, Married girl, Awache).

For younger adolescent girls, menstruation is often a source of fear and shame, as they fear mockery: “If the blood comes out, they will mock them and chase them away” (Body Mapping, Girl, Garare). Yet for the broader community, it acts as a silent, immediate alarm. Mothers actively teach girls to manage menstruation secretly: “We advise them to be clean and take care of themselves to the point where someone couldn't even understand that they are menstruating” (Social norms, Mothers, Maiguije). Ultimately, body mapping participants explicitly link this physical maturation to marital readiness, noting, “She has reached marriageable age”.

This gendered asymmetry is deeply internalized. Mothers reinforce a distinct cultural flexibility for boys’ maturity, stating “a boy can go up to 30 years old in celibacy and it doesn't cause any problem” before marrying (Social norms, Mothers, Awache). Boys are expected to work to prepare for marriage; as fathers explained, when a boy reaches puberty, the immediate priority is to find him an apprenticeship, such as in sewing or a mechanics garage, so he can eventually support a family (Social norms, Fathers, Magaria). For girls, adolescent boys explicitly view female menstruation as a signal of marital “ripeness,” noting that “if someone is pubescent we have the idea that she has reached the age of marriage… she can have children” (Body Mapping, Boy, Garare).

### Management of shame through risk mitigation

Participants described how the biological milestone of menarche frequently elicits profound parental anxiety over shame, motivating a preventive strategy enforced by fathers and community leaders, who act as the primary reference groups demanding premarital chastity. To mitigate this anticipatory shame, parents frequently deploy negative sanctions as pre-emptive measures to control girls’ mobility, including enforcing strict curfews and utilizing harsh corporal punishment. Illustrating how staying out late leads to physical punishment due to these fears, one participant explained: “Parents hit out of fear that she might pop over to the boys’ place or follow bad girls who will mislead her… and get pregnant out of wedlock” (IDI, Girl, Oura). A girl who challenges this norm by contracting an out-of-wedlock pregnancy is perceived as facing negative sanctions, including absolute social rejection. Community leaders emphasized the severity of this punishment: “If she catches a pregnancy, there can be a problem regarding the social rejection of the girl and even becoming the most hated in the community…” (Social norms, Community leaders, Garare). The fear of community gossip and judgment is so immense that fathers may entirely disown a transgressing daughter to distance themselves from the shame: “It is a way of denigrating her father's image in the eyes of the world… he rejects her because he considers her the source of his social defeat. So, it is better to lose the daughter” (Social norms, Mothers, Awache).

While navigating these pressures, data indicate that some girls independently seek out contraceptive methods to mitigate risk. Rather than merely waiting for a marriage arrangement, mothers noted that girls who wish to continue their education proactively seek out contraceptives to buy time. As one group of mothers explained: “The girls who like to study, it is they themselves who go to get contraceptive methods until they finish their studies” (Social norms, Mothers, Awache). These contraceptives are used secretly to buy time for girls to finish school or find a husband, offering a temporary sense of security against the stigma of premarital pregnancy.

Ultimately, however, child marriage was commonly described as a primary tool to permanently transfer the reputational risk away from the parents and onto the husband's household. Mothers explicitly frame marriage as a preventive strategy against social disgrace to secure the positive reward of a preserved family reputation and a “clear conscience”. As one noted: “We prefer to give her in marriage before she gets pregnant by a third party… if we give her in marriage, then we will have a clear conscience” (Social norms, Mothers, Maiguije). Operating as a protective reference group, mothers actively seek traditional protections first: “As soon as a girl is pubescent, we do everything to protect her against anything bad that can happen to her, with amulets and by giving her traditional pharmacopoeia products so she doesn't get pregnant” (Social norms, Mothers, Maiguije). This strategy is reinforced by broader community discourse, where the fear of uncontrolled adolescent sexuality is normalized. This profound fear of an out-of-wedlock pregnancy—often framed as bringing a “bastard” into the home—acts as a prominent catalyst for preventive child marriages. The stigma is immense: “If an unmarried girl or a divorcee has an abortion, the population itself will talk a lot about the negligence of her parents” (Social norms, Fathers, Awache). Furthermore, an unmarried adolescent who becomes pregnant and seeks a clandestine abortion is perceived by the community as committing “two crimes”—the out-of-wedlock pregnancy and the abortion itself—which frequently results in her being permanently expelled from the family home. In this context, marriage serves as a tool to transfer the shame risk away from the parents. As one participant observed, parents fear premarital relationships, so they marry their daughters off early so that “whatever she thinks of doing, she will do it at her husband's house; the parents avoid shame in the village” (IDI, Married girl, Oura).

Importantly, participants indicated that this structural policing does not end with the wedding; rather, post-marital violence operates as a direct continuation of gendered normative control. Physical violence serves as a primary negative sanction for any behavior that challenges the norm of chastity or obedience. Once married, the enforcement of these norms is coordinated between two powerful reference groups: the husband and the parents. If an adolescent wife challenges the norm of obedience, husbands will either apply physical sanctions directly or summon her parents to do so. As one unmarried boy detailed: “These are adolescents who do not respect their husbands. Given their importance and value in the eyes of the population, these husbands summon the girl to her parents so that they draw her attention. The parents on their side, since this is not what they taught their daughter, they interrogate her and hit her. But some husbands do not tolerate being devalued by their wives… they hit her” (IDI, Boy, Oura). Furthermore, challenging these strict family norms carries the long-term negative sanction of losing the family as a protective reference group entirely. As one adolescent girl explained, defying the family means facing future hardships alone: “Because anyone who dares to do something that goes against her family will face multiple negative consequences. For example, if after the marriage she encounters a marital problem, they will say, ‘Here is what finally happened to you, had you listened to us, it would not have been like this’” (IDI, Girl, Garare).

### The intersection of social norms and structural factors

Ultimately, participants noted that this preventive strategy is frequently compounded by economic vulnerability, where early marriage is utilized as a strategy to alleviate household expenses. The normative pressures to protect family honor are inextricably linked to the financial reality of poverty; parents expressed fear that financially unsupported girls may engage in sexual relationships, thereby risking the family's reputation. Mothers explicitly linked this fear to the lure of financial gifts from older men: *“Girls today are attracted by things, by money… it is the older men who spoil the girls by giving them money, and it is through this that they betray them. So, we are forced to panic as soon as our daughters start their periods” (Social norms, Mothers, Maiguije).*

Mothers perceived that the lure of minor financial gains strongly influenced girls’ behavior: “Because of a phone, 500 CFA francs, or 1000 CFA francs, your daughter gives herself to such a relationship, something she can spend in a short amount of time!” (Social norms, Mothers, Garare). While some girls may attempt to prevent pregnancy using modern contraceptives, adolescent accounts focused heavily on the severe social penalties applied to those who become pregnant out of wedlock. Describing the social rejection experienced by these girls, one participant stated simply: *“Some girls use modern contraceptive methods, so it is not so easy to identify them, but among those who do not use any method, some of them get pregnant, resulting in out-of-wedlock pregnancies… She was looked upon as less than nothing” (IDI, Girl, Oura).*

Furthermore, participants recognized that poverty can drive women to engage in transactional sex as a survival mechanism, particularly when husbands migrate for work. Fathers acknowledged this structural desperation: “If the woman has nothing to feed herself, she is forced to go to someone even if they will have a sexual relationship to give her something” (Social norms, Fathers, Awache). Community leaders corroborated that this is a systemic issue born of economic necessity: “You know, most often it is poverty. A woman with children without food and her husband is in exodus, so obligatorily she will give herself to such an act to be able to feed the children” (Social norms, Community leaders, Magaria).

Summarizing how poverty accelerates early marriage, one boy succinctly noted, “Parents are unable to take care of their children… so they decide to give her in marriage” (IDI, Boy, Oura). Preventive child marriage was thus commonly described as a dual-purpose strategy, simultaneously functioning as a perceived moral safeguard against the shame of premarital sexuality and providing immediate economic relief for impoverished households.

## Discussion

This study set out to explore the bio-social and cultural drivers of child marriage in rural Niger, focusing on how communities manage the perceived risks of adolescent female sexuality. As vividly articulated across our diverse participant groups—from the anxieties voiced by mothers and community leaders to the fatalistic acceptance expressed by adolescent girls, our findings indicate that child marriage in this context was commonly described as a mechanism to manage social shame through proactive risk mitigation. This practice is triggered by the biological onset of menarche, driven by profound parental anxieties over anticipatory shame, and exacerbated by household poverty. By applying a socio-cultural lens to the intersection of gender norms and extreme poverty, this study contributes to a more nuanced understanding of how biological maturation and structural vulnerabilities collaborate to sustain child marriage, highlighting both the constraints placed on adolescent girls and the complex agency exercised by their mothers.

### The “puberty deadline” and the gendered construction of adulthood

The findings suggest that menarche is culturally interpreted not merely as a biological transition, but as a social marker of sexual vulnerability that accelerates girls into socially regulated adulthood.

This aligns with recent scholarship emphasizing the concept of “marriageability,” wherein social norms determine what it means to be ready for marriage and what the consequences are if that status is compromised (15, 17, 19). However, our findings introduce the unique conceptual contribution of the “puberty deadline,” which extends beyond general marriageability by identifying a specific, socially enforced temporal expectation: a narrow, rigid window immediately following menarche during which a girl is expected to marry. This temporal norm is actively upheld by distinct reference groups: peers enforce the timeline through the threat of mockery, mothers and maternal aunts manage the immediate physical transition and enforce secrecy, and fathers and community leaders orchestrate the marriage to prevent anticipated family shame. Furthermore, participants indicated that surpassing this perceived deadline carries severe social sanctions, resulting in the girl being stigmatized by the community and labeled as an outcast or “old maid”. In rural Niger, a girl's physical maturation acts as a silent alarm, initiating an acute cultural panic surrounding her sexuality.

Crucially, our data highlight a stark gender asymmetry in the transition to adulthood. While puberty immediately qualifies girls for marriage to prevent sexual transgression, adolescent boys are granted a prolonged, socially sanctioned transition period. Upon reaching puberty, boys are encouraged to pursue apprenticeships and economic independence before marrying in their late teens or twenties. This discrepancy reflects deeply entrenched patriarchal narratives that view female sexuality as an inherent risk that must be controlled, whereas male sexuality and maturity are linked to economic provision (1, 14). This double standard means that while boys are actively transitioned into the public economic sphere, girls are pushed into the private domestic sphere, sharply curtailing their educational and developmental trajectories (16, 18). Quantitative research from rural Niger further demonstrates that marrying as a very young adolescent places girls in an extraordinarily disadvantaged position regarding household agency. Alarmingly, adolescent wives frequently internalize these inequitable gender norms, reporting high satisfaction despite their almost complete exclusion from economic, mobility, and health-care decisions (15, 27). This underscores how the abrupt transition to marriage heavily suppresses female agency and normalizes male dominance in decision-making.

### Anticipatory shame, premarital pregnancy, and risk mitigation

Our findings indicate that anticipatory shame was frequently cited as a prominent motivating factor for enforcing the puberty deadline. Operating as a broader family and community process, parents and extended networks described utilizing child marriage as a proactive measure of risk reduction to protect family honor from the widely feared social stigma of premarital sex and out-of-wedlock pregnancy. This corroborates evidence from other sub-Saharan African contexts, where the threat or reality of premarital pregnancy acts as a primary driver of child marriage (16, 23). Similarly, recent qualitative research from Mozambique suggests that the cultural significance placed on a girl's first menstruation, combined with the intense social pressure to avoid the dishonor of an out-of-wedlock pregnancy, strongly influences families to utilize child marriage as a protective strategy against shame and poverty (20). In communities where an unmarried pregnant adolescent faces ostracization or expulsion, marriage effectively transfers the “risk” of her sexuality from her parents’ household to her husband's (5, 14). This practice is driven by a tragic misconception of the need to “protect” a girl's sexuality (1, 6). Paradoxically, by rushing girls into marriage to avoid the social shame of premarital pregnancy, parents expose their physically immature daughters to catastrophic reproductive health risks, including obstructed labor and obstetric fistulae (11, 13).

However, our study also uncovers adolescent reproductive agency as a critical and underexplored dimension of this risk mitigation. While overarching patriarchal norms dictate marriage as the ultimate solution, data indicate that girls themselves actively seek alternative strategies to protect their futures. The finding that girls who wish to continue their education independently and covertly access contraceptive methods reveals a complex negotiation of social norms. It is crucial to distinguish between mothers’ agency in navigating social norms—often by tacitly accepting these practices to secure a “sense of security” against pregnancy-related shame—and the girls’ own reproductive agency, consent, and autonomy in seeking out the services. Girls use family planning not necessarily to challenge the institution of marriage, but to buy time for their daughters to finish school and to secure a “sense of security” against pregnancy-related shame. This resonates with recent research highlighting how family planning beliefs and child marriage intersect, showing that covert contraceptive use is a vital, albeit constrained, expression of female agency in highly patriarchal settings (11, 16, 22).

### The intersection of social norms and structural factors

The social imperative to manage shame cannot be decoupled from the structural vulnerabilities characterizing these subsistence farming communities. Our data suggest that the moral panic surrounding female sexuality intersects directly with poverty, often turning the puberty deadline into a perceived strategy for economic coping. For impoverished families unable to support their children, a post-pubescent daughter is often viewed as both a moral liability and a factor of household poverty. Preventive child marriage is thus perceived as a dual-purpose strategy: families use it in an attempt to safeguard family honor while seeking perceived financial relief. These findings echo ethnographic research from other sub-Saharan contexts, such as Uganda, which illustrates how poverty drives families to treat daughters as a critical survival strategy, even directly exchanging them for livestock or bride wealth to alleviate economic hardship (3, 14, 20).

As demonstrated by the direct accounts of parents and adolescents navigating extreme poverty, this intersection underscores that child marriage is rarely a choice made in a vacuum; rather, it operates within a “child marriage economy” where social norms combine with structural factors such as poverty to drive girls’ risk and vulnerability to child marriage (3, 5). As noted in the broader literature, poverty accelerates the perceived “readiness” for marriage, as families fear that financially unsupported girls may engage in transactional sex to meet their basic needs, further risking the family's reputation (14, 16). Therefore, interventions that view child marriage solely as a harmful cultural tradition, without addressing the underlying economic desperation that sustains it, are likely to fall short (4, 28).

### Limitations

This study has several limitations. The findings are contextually grounded in selected rural and peri-urban Hausa-speaking, Islamic, agrarian communities in the Maradi region, which may limit their transferability to other sociocultural settings in Niger. The cross-sectional nature of the data collection precludes establishing temporal or causal relationships between menarche, anticipated shame, household poverty, and marriage decisions. The sensitive topics of adolescent sexuality, menstruation, contraception, abortion, and violence may have led to socially desirable responses. While participatory visual methods, vignettes, and individual interviews were employed to encourage disclosure, the focus group discussion setting may have reinforced dominant community norms and discouraged dissenting or personal accounts. Some findings reflect participants’ perceptions of expected social consequences rather than directly observed or personally experienced practices. Additionally, transcription and translation from Hausa into French and then into English may have diminished some culturally specific meanings, particularly regarding shame, maturity, and marriageability. Finally, purposive sampling of socio-demographically similar communities may have limited the representation of divergent perspectives and variation across participant subgroups.

### Conclusion and implications for practice

The persistence of child marriage in Niger is sustained by a complex web of biological triggers, cultural anxieties over female sexuality, and economic constraints. The findings of this study suggest that relying solely on legal bans or abstinence-only education will likely be insufficient to eliminate the practice. As evidence on effective strategies to prevent child marriage indicates, successful programs often address the root social determinants by enhancing girls’ human capital and economic opportunities (8, 19, 28).

For social and behavior change interventions to be effective, they should engage directly with the cultural narratives of “shame” and the gendered constructions of maturity. As evidenced by interventions across Africa, engaging community and faith-based gatekeepers is widely recognized as a critical component to successfully shift these deeply entrenched social norms (29). When expanding access to youth-friendly sexual and reproductive health services and family planning, interventions should prioritize girls’ informed choice, consent, confidentiality, and reproductive autonomy. While the covert desires of mothers to protect their daughters can be a supportive factor, it is vital that programs center on the adolescent's own agency rather than equating maternal action with girls’ autonomy. Furthermore, the design and implementation of these interventions should explicitly and meaningfully include adolescents themselves. Finally, structural interventions such as conditional cash transfers or support for girls’ secondary schooling can help to offset the economic vulnerabilities that often pressure parents to consider the “puberty deadline” as a financial coping mechanism (5). Comprehensive approaches that empower girls with information while simultaneously providing economic support and incentives to their families have shown promise in delaying marriage (8, 30). Only by addressing the bio-social fears and economic realities that drive preventive child marriage can policymakers and practitioners foster genuine, culturally informed normative change.

## Data Availability

The raw data supporting the conclusions of this article will be made available by the authors, without undue reservation.
